# Epidemiology and Seasonality of Childhood Respiratory Syncytial Virus Infections in the Tropics

**DOI:** 10.3390/v13040696

**Published:** 2021-04-16

**Authors:** Manika Suryadevara, Joseph B. Domachowske

**Affiliations:** Department of Pediatrics, SUNY Upstate Medical University, New York, NY 13210, USA; suryadem@upstate.edu

**Keywords:** respiratory syncytial virus (RSV), tropics, seasonal epidemiology, infant lower respiratory tract infection

## Abstract

Infections caused by respiratory syncytial virus (RSV) are a major cause of morbidity and mortality in young children worldwide. Understanding seasonal patterns of region-specific RSV activity is important to guide resource allocation for existing and future treatment and prevention strategies. The decades of excellent RSV surveillance data that are available from the developed countries of the world are incredibly instructive in advancing public health initiatives in those regions. With few exceptions, these developed nations are positioned geographically across temperate regions of the world. RSV surveillance across tropical regions of the world has improved in recent years, but remains spotty, and where available, still lacks the necessary longitudinal data to determine the amount of seasonal variation expected over time. However, existing and emerging data collected across tropical regions of the world do indicate that patterns of infection are often quite different from those so well described in temperate areas. Here, we provide a brief summary regarding what is known about general patterns of RSV disease activity across tropical Asia, Africa and South America, then offer additional country-specific details using examples where multiple reports and/or more robust surveillance data have become available.

## 1. Introduction

Worldwide, acute respiratory tract infections (ARTIs) are a leading cause of morbidity and mortality among children less than five years of age [1,2]. Globally, an estimated 650,000 children less than five years of age die from lower respiratory tract infections (LRTIs) every year [2]. Those living in low income countries and those who are exposed to low socioeconomic environments are the most vulnerable [2,3]. Infections caused by respiratory syncytial virus (RSV) are a major contributor to the morbidity and mortality associated with childhood respiratory tract infections [2,3,4,5,6,7]. Like influenza viruses and several other respiratory viral pathogens, RSV is known to cause seasonal outbreaks every year. While the timing, peak and duration of these outbreaks vary geographically, they tend to be somewhat predictable by location from year to year. Understanding the seasonal patterns of region-specific RSV activity is important to guide resource allocation for existing and future treatment and prevention strategies.

However, merging evidence suggests that tropical regions of the world may not have the same predictable, patterned seasonal peaks or outbreaks of respiratory viral infections [8,9,10]. Across many large, heavily populated regions of the tropics, pathogen-specific data are sparce or nonexistent. The lack of clearly defined temporal patterns of viral activity makes it difficult to optimize the implementation of effective public health measures such as annual influenza vaccination [11,12,13] or scheduling high risk infants to receive passive immunotherapy for protection against RSV infection.

Decades of excellent surveillance data are available that describe the annual patterns of infant and childhood RSV infection in the developed countries of the world including most of Europe and North America, Australia and the developed nations and special administrative regions of Asia (i.e., Israel, Japan, South Korea, Singapore, Taiwan, Hong Kong and Macau). Such surveillance data are incredibly instructive in advancing public health initiatives in those countries. With few exceptions (Singapore, Hong Kong, Macau and the northernmost area of Australia), these developed nations are positioned geographically across temperate regions of the world. Available RSV surveillance data from the tropics are more scarce, and when available, are still largely lacking in sufficient longitudinal study to permit truly robust analyses of seasonal patterns and climate influences over time. However, existing and emerging data collected across tropical regions of the world do indicate that patterns of infection are often quite different from those so well described in temperate areas.

Simply defined, the tropics are those areas of the Earth where the Sun is positioned directly overhead at least once a year. The Earth’s axial tilt defines those northern and southernmost geographical coordinates at the latitudes referred to as the Tropic of Cancer (23.43° N) and the Tropic of Capricorn (23.43° S). Between these latitudes lies an area that includes approximately 40% of the Earth’s surface and 36% of its total landmass. Currently, it is estimated that 40% of the world’s population lives in the tropics. Unlike the four seasons that occur each year across temperate zones of the world, many tropical areas experience fairly predictable meteorological cycles referred to as their dry and wet (rainy) seasons.

Although the collection and reporting of RSV surveillance data from some tropical areas of the world has improved substantially in recent years, far less is known about the seasonality of RSV disease in tropical countries compared to those with temperate climates. Here, we provide a brief summary regarding what is known about general patterns of RSV disease activity across tropical Asia, Africa and South America, then offer additional country-specific details using examples where multiple reports and/or more robust surveillance data have become available.

## 2. RSV Disease Activity across Tropical Asia

RSV is among the most common causes of lower respiratory tract infections in children across Asia [14,15]. In some studies, RSV detection was more than 3 times greater than the next leading pathogen [14]. Reported RSV positivity rates in tropical Asian countries range from 9% to 50% depending on the geographic location and sample population (Table 1) [16,17,18,19,20,21,22,23,24,25,26,27,28,29,30,31,32,33,34]. Not unexpectedly, an increased risk for RSV infection has been noted in infants with chronic lung disease, neurodevelopmental conditions and congenital heart disease, with repeated infections associated with asthma and atopy among the children and their families [15].

In the tropical regions of Asia, RSV epidemics, defined as a sudden increase in the number of infection cases above what is normally expected in the area, vary by geographic location and regional altitude, with each season most typically starting along the coast and spreading inland [15]. In general, RSV infections are seen year-round with peak activity occurring during rainy seasons of the tropical island nations including Singapore (1.4° N latitude), Taiwan (23.7° N), and Malaysia (4.2° N) [15,35]. In contrast to temperate regions, where there is a clear winter seasonality to annual RSV epidemics, trends across tropical countries are different from region to region despite geographical proximity [35]. Where surveillance data are available, peak RSV activity across the region can occur in January and February (Nepal), July to November (Thailand), December to February (Bangladesh) or February to May (Indonesia) (Table 1). Regional differences in climate patterns, elevation above sea level and outdoor air quality have been used, at least in part, in attempts to explain the wide variations described in RSV seasonality. For example, in Malaysia (4.2° N), RSV disease activity is positively correlated with mean daily rainfall, and negatively correlated with relative humidity and mean daily temperatures [22]. In contrast, RSV disease activity in Indonesia (0.8° S) shows a positive correlation with mean daily rainfall, relative humidity and mean temperature [17].

### 2.1. RSV in Philippines

The Philippines is an archipelagic country consisting of over 7000 islands located 12.9° N of the equator in the western Pacific Ocean. The climate is tropical, with high temperatures, humidity and rainfall. The mean annual temperature is 26.6 °C, with January and May being the coolest and warmest months, respectively. Regional altitude, more than latitude, explains the wide temperature variation, with cooler temperatures found at higher elevations. The rainy season occurs from June to November. The remainder of the calendar year is subdivided into cool (December to February) and hot dry seasons (March to May) [36].

Excellent surveillance across the Philippines consistently shows RSV ranking as one of the top two most commonly detected respiratory viruses each year, and as the leading cause of hospitalization among infants and young children [25,26,27,37,38,39,40]. Furuse et al. found that nearly 24% of all RSV-infected children visit a medical facility during their infection, with 2% requiring hospitalization [38]. Reported RSV positivity rates range between 12 and 27% by location, but the highest rates of detection and incidence of severe disease are consistently seen among children less than 2 years of age [25,26,27,37,38]. In addition to young age, other risk factors for the development of severe RSV included a lower educational level of caregivers and underlying malnutrition [26,39]. Like other parts of the world, transmission of RSV infection within households is well documented. Surveillance efforts in the Philippines have shown that up to 30% of infants infected with RSV acquire the infection from an older child living in the same household [41].

In Baguio City (population ~350,000; elevation 4800 ft), the average RSV incidence rates are reported at 1.4 per 1000 individuals, with the highest age-group-specific incidence rates of 21.7–38 per 1000 individuals occurring in infants 6–23 months of age [40]. Among children less than 6 mos of age, mean hospitalization rates for RSV were between 2 and 8-fold higher than for influenza. Similar incidence rates for RSV lower respiratory tract infection were found among those living on the small island province of Biliran [39].

RSV epidemics have been shown to be impacted by a variety of meteorologic factors that influence the seasonality of infection. Year to year, viral activity across the Philippines is predictably most pronounced between September and December, with some regional variability. Peak infections tend to occur during the rainy months of October and November [25,37,40,42,43]. The interplay between meteorology, host and socioeconomic factors is notable as the rainy season has been shown to be associated with more advanced malnutrition, a known risk factor for severe RSV disease [43]. Paynter et al. found that seasonal malnutrition precedes the seasonal peak of both RSV infection and pneumonia incidence in infants by approximately 10 weeks [44].

### 2.2. RSV in Singapore

The nation of Singapore is an island nation that includes the main Singapore Island and 60 smaller islets. Located between Malaysia and Indonesia, it sits just one degree of latitude north of the equator. The climate is tropical, with high rainfall, high temperatures and high humidity year-round. Mean daily temperatures range between 23.9 °C and 33 °C, with the highest monthly temperatures seen in May and June and the lowest in December and January. There are two monsoon seasons that stretch from December to early March and from June to September [45].

RSV causes a significant disease burden in Singapore. It has been reported as the most commonly detected virus among children hospitalized with a respiratory tract infection, with the highest positivity rates among children younger than 2 years of age [29,46,47]. Like other regions across the globe where accurate surveillance data are tracked, children in Singapore who have underlying hemodynamically significant heart disease, immunodeficiency and/or metabolic disease are at an increased risk for RSV-related death [48].

Between 2005 and 2013, RSV accounted for 42% of all bronchiolitis and pneumonia among children younger than 30 months of age who were hospitalized in Singapore [48]. Tam et al. reported an estimated 170 primary care consultations for RSV per 1000 child-years among children younger than 6 months of age [49]. The healthcare cost attributable to RSV infection in Singapore is estimated at $4.7 million USD annually [49]. RSV activity is detected year-round in Singapore with most epidemics occurring between May and September [29,49].

## 3. RSV Disease Activity across Tropical Africa

RSV infection causes substantial morbidity and mortality in children worldwide, including those who live in areas of tropical Africa. While the pooled prevalence of RSV infection among individuals with acute respiratory tract infections across several tropical regions of Africa where surveillance data are available is reported at 14.6%, there is a wide variation in infection prevalence regionally [50]. Table 2 offers a summary of available RSV epidemiology in tropical African countries, demonstrating a very wide range of RSV positivity rates, between 2.7% and 47% [51,52,53,54,55,56,57,58,59,60,61,62,63,64,65,66,67,68,69,70]. The reported incidence of RSV-associated lower respiratory tract infection differs widely by country, with extremes ranging from 0.46 (Malawi) to 112 (The Gambia) per 1000 population [55,67]. The wide ranges reported for both RSV positivity rates and the incidence of lower respiratory tract infections from one region to the next likely reflect, in part, regional differences in the quality of surveillance data due to differences in methodology, reporting and available diagnostic resources. Region to region variations in climate, elevation, outdoor air quality, poverty levels, civil unrest, maternal and infant nutrition and other underlying host factors also likely impact rates, severity and timing (seasonality) of RSV outbreaks. Across all of sub-Saharan Africa, RSV has been estimated to account for 13.4% of all cause deaths among children less than 5 years of age [71]. This already alarmingly high percentage may under-estimate RSV’s full impact, as underlying causes of infant deaths that occur in rural areas and across communities without access to health care may never be determined or reported at all. Worldwide, RSV disease creates a substantial economic burden. In Malawi, the mean cost per RSV episode is estimated at $62.26 USD per inpatient case. At first glance, the cost may seem trivial, but considering the economy of the nation, $60 translates to nearly one third of the total average monthly household income [72].

Consistent with worldwide data, RSV infection in Africa is more frequently identified in children than in adults [50,73]. Identified risk factors for RSV infection across this region include young age, not breastfeeding and day care attendance [52,53,55,56,57,61,69]. In Malawi, where HIV infection is highly prevalent, no differences were identified in the rates of RSV-associated severe acute respiratory tract infection between children with or without HIV infection [67].

Table 2 provides a summary of published data from studies describing RSV epidemiology in tropical African countries. As seen in the table, the timing of RSV disease activity across this area varies from country to country. Specifically, the cyclical patterns of RSV infection occurring during the rainy seasons across Madagascar (18.8° S), Burkina Faso (12° N), Mozambique (19° S) and Réunion Island (21° S) are not described to occur in other regions of tropical Africa. For example, in Ghana (7.9° N), one report describes peak RSV activity as occurring between June and December, yet the longest and heaviest rainy season occurs between April and June. Similarly, available surveillance data from The Gambia (13.4° N) and from Kenya (0°) have not shown consistent clear associations between RSV disease activity and rainy seasons (Table 2) [73].

### 3.1. RSV in Kenya

Kenya is positioned in East Africa, bisected by the equator. Its coastline runs for 1400 km along the Indian Ocean. Along the coast, the climate here is tropical, with temperatures averaging 27 °C. The country is mountainous with a peak elevation of more than 17,000 ft. Changes in altitude lead to regional climatic variations, including cooler average daily temperatures. Two rainy seasons occur each year, one from April to June, the other from October to December [74].

Nationally, RSV surveillance work has shown positivity rates ranging between 3.2 and 14% [58,59,60,61]. A recent active surveillance household study found RSV to be the fourth most common virus detected, with a positivity rate of 3.2% [58]. Over the course of the study period, RSV was detected in 85.1% of households, with an individual risk of RSV infection estimated at 37.1% [58,75]. RSV infection was detected more frequently in individuals diagnosed with lower compared with upper respiratory tract infections [59]. Asymptomatic RSV infection (presumably re-infection) was associated with older age, larger household size and infection with RSV subtype B [75]. In homes where a clear index case could be identified, the main source of infant infection was transmission from a toddler or school-aged child also residing in the household [76].

Countrywide, excellent surveillance efforts have estimated the incidence of acute respiratory tract infection due to RSV at 767 per 100,000 child years among children younger than 5 years of age. Infections caused by RSV account for 3% of all outpatient visits and 8% of all acute respiratory tract infections [59]. Mean annual mortality rates associated with RSV infection are estimated at 17.1 and 7.3 per 100,000 person years for all cause excess mortality and for respiratory deaths, respectively. The highest fatality rates due to RSV infection occur among children younger than 5 years old [77].

Available data from Kenya do not consistently demonstrate an association between RSV seasonality and rainfall, relative humidity or mean daily outdoor temperature. RSV seasons in Kenya are consistently long, and without clear climate influences [78]. Seasonality in the coastal counties of Kilifi (January to May, with a peak in March and April) and Siaya (February to September, with a peak in May to July) overlapped very little despite similar climates [58,60,75]. On the other hand, infections in Nairobi, the inland country capital, showed no clear pattern of RSV seasonality, with RSV illnesses reported year-round [60].

### 3.2. RSV in Madagascar

Madagascar is an island country positioned in the Indian Ocean 18.8° S of the equator off the east coast of Africa. The climate here is sub-tropical to tropical, with hot rainy summers (November to March) and cool, dry winters (May to October). Average annual temperatures range between 23 °C and 27 °C along the island’s coastline, and between 16 °C and 19 °C at the higher mountainous elevations. Changes in elevation account for regional temperature variations [79]. Not surprisingly, RSV is among the most common viruses detected in published surveillance studies, showing positivity rates ranging between 11.8% and 43.5% [63,64,65,66]. Mean annual hospitalizations due to severe RSV lower respiratory infection among all age groups are estimated at 11,768 per 100,000 population, with the highest rates occurring among children younger than 5 years of age [64,66,80]. RSV infection peaks once a year in Madagascar, resulting in annual epidemics, but the timing of seasonal epidemics varies from year to year, most often occurring during rainy summer seasons [64,65,66,80].

## 4. RSV Disease Activity across Tropical South America

In South America, RSV is among the most common respiratory virus detected in individuals presenting with acute respiratory tract infections, and is a major cause of infant and young child hospitalizations [81,82,83]. Reported RSV positivity rates range between 3% and 46.3%, depending on the specific cohort studied, with the highest disease burden detected in children younger than 1 year of age (Table 3). In Bolivia, the average incidence of hospitalizations due to RSV-associated severe acute respiratory infections is estimated at 9 per 100,000 hospitalizations [84]. In equatorial Brazil, RSV was shown to have a more significant clinical impact than influenza, including illness caused by the 2009 pandemic influenza AH1N1 strain [85]. In general, risk factors associated with RSV infection include young age, decreasing gestational age, presence of a smoker in the household, living at higher elevations, day care attendance and lower paternal educational levels [82,86].

Reports from tropical countries in South America that describe results from RSV surveillance studies performed for more than one season are scarce. Where available, such studies have shown that RSV seasonality varies by country and even by geographic regions of the same country. Since most reports include small numbers collected over a period of 1 year or less, comparisons of perceived trends or patterns across different countries or regions will need to wait until multi-year data have been collected from larger sized cohorts. In Suriname (3.9° N), RSV activity is detected all year round, without clear evidence of seasonal peaks. In some parts of Brazil, infection rates have been reported to peak during the rainy season, lending positive correlations between virus activity and rainfall, lower maximum outdoor temperatures and lower mean daily wind speed [82,87,88]. Since all 3 meteorological parameters show such patterns during Brazil’s rainy season, it’s not possible to determine which of them, if any, are influencing RSV seasonality.

### 4.1. RSV in Colombia

Colombia is positioned at 4.6° N latitude at the northwest corner of South America with sections of coastline along both the Pacific Ocean and the Caribbean Sea. The coastal climate is tropical. Changes in elevation from sea level to interior geographical points in the Andes Mountain range that reach nearly 19,000 ft contributes most to the observed variations in regional mean temperatures. Mean annual temperatures range between 14 °C in the mountains to 27 °C across the tropical savannah and coastline [98]. Each year, the rainy seasons span between April and May and again between October and November with the dry seasons spanning the months of December and January and the months of July and August.

RSV positivity rates among children younger than 1 year of age with lower respiratory tract infection have been reported to range between 30 and 46% [92,93,94]. Risk factors associated with severe RSV infection are similar to those described in other parts of the world, including young age, neurologic co-morbidities, chronic lung disease of prematurity and congenital heart disease [93]. The median total cost for an infant hospitalized with RSV in a general pediatric ward is estimated at $518 USD, while the estimated mean cost for a single RSV-related hospitalization that includes pediatric intensive care unit is $2749 [99]. Total costs of RSV infection among all children younger than 2 years in Colombia has been estimated at more than $64 million USD [100]

RSV infection is routinely detected throughout the year in Colombia. Rates of infection and related hospitalizations peak between March and May, a period that immediately precedes and then spans the first of the two rainy seasons [93,94,101,102,103]. Monthly average temperature and rainfall have been shown to correlate with RSV disease activity across the region [94,103].

### 4.2. RSV in Ecuador

Ecuador, positioned in the northwest of South America, spans the equator. Its Pacific coastline spans more than 2000 km. Elevations climb to more than 20,000 ft in the Andes Mountain range moving toward the country’s interior. The climate along the coast is tropical, while patterns of daily temperatures and rainfall at higher elevations are more typical of those encountered in temperate climates. Along the coast and across the lower elevations the rainy season typically spans January to mid-April (longer in some areas), with total annual rainfall of approximately 489 mm, most of which falls over a period of just a few weeks. The period between April and December sees little or no rainfall at all. Rain occurs year-round in Quito with annual total amounts totaling more than 1273 mm.

Published surveillance data on RSV infection in Ecuador are limited to reports describing the causes of severe lower respiratory tract infection among children less than 5 years of age who are hospitalized in Quito (elevation 9350 ft) [95]. RSV detection was reported to be highest among children younger than 1 year, with a decreasing rate of detection with increasing age [90]. A second report confirmed that children younger than 1 year of age were more likely (1.7-fold) to have RSV identified compared with children 1 to 5 years of age [96]. The peak period for childhood RSV-associated hospitalizations was seen during the months of January and February.

In July 2018, we launched a 5-year prospective surveillance study to describe the causes of acute respiratory tract infections among outpatient Ecuadorean children less than 5 years of age. Study sites include busy outpatient pediatric practices along the coast (Machala), and in the nation’s capital city of Quito. The study sites are separated by 230 miles and differ in elevation by more than 9300 feet. Children younger than 5 years of age who are diagnosed clinically with acute respiratory tract infection are eligible for enrollment. After obtaining informed consent, demographic and illness-specific data are recorded and a nasopharyngeal swab collected for diagnostic testing using the BioFire^®^ FilmArray^®^ Respiratory Panel (Salt Lake City, UT, USA). Between July 2018 and June 2020, 850 subjects were enrolled, 491 from Machala and 359 from Quito. During these first 2 years of surveillance, 12% of all samples were positive for RSV, ranking it second to rhinovirus/enterovirus as the most commonly detected pathogen both overall and by site of enrollment. The seasonal epidemiology of illness attributed to RSV infection differed substantially between the two study locations, with activity in Machala starting during February, lagging Quito by nearly three months, where RSV activity began during each preceding November. To date, nearly all of the RSV infections detected at the Machala site occurred between February and April, a period of time corresponding to the rainy season ([104] and unpublished data). In Quito, both RSV seasons studied to date began in November and peaked in February, a pattern typical of the seasonal patterns described in temperate areas of the world including the United States and Europe ([104] and unpublished data).

Globally, RSV infections remain a leading cause of infant and childhood morbidity and mortality. A safe and effective vaccine remains elusive and therapeutics that improve the course of infection have yet to be identified. Maternal immunization approaches, where investigational RSV vaccines are administered during pregnancy, have recently emerged as promising strategies [105,106] to prevent RSV infection in their newborns. Unfortunately, enthusiasm for this approach was dampened with the report that the first completed phase 3 clinical trial failed to meet its primary endpoint [106]. However, substantial progress is being made, with novel extended half-life monoclonal antibodies being developed for passive prevention of RSV infection during infancy. Results from a pivotal single-dose, randomized, placebo-controlled phase 2b registration study (NCT02878330) in preterm infants born between 29 and 35 weeks gestational age showed a 70% reduction in medically-attended RSV lower respiratory tract infections 70.1%) and a 78% reduction in RSV-associated hospitalizations compared with a placebo. The side effect profiles were similar for subjects randomized to receive either the monoclonal antibody or placebo [107,108]. The program has already advanced to a phase 3 (NCT03979313) randomized, placebo-controlled trial that includes term infants entering their first RSV season [109,110,111]. The ultimate goal is use of a single, fixed dose for all newborns and infants entering their first RSV season. To realize its full potential to reduce the morbidity, mortality and misery caused by RSV on a global scale, robust and reliable regional surveillance data on the seasonality of infection are needed so that ministries of health and public health programs can determine the ideal timing of administering the dose. Equally important considerations include cost, production and availability and that each aligns with the capacity, need and demand from stakeholders across the globe. The World Health Organization, national health ministries and the public health systems they support must collaborate closely with the manufacturer(s) and the multiple layers of infrastructure needed to distribute, store and deliver passive RSV protection to the world’s birth cohort each year. Ideally, administration of the extended half-life monoclonal antibody across each region will be prioritized just prior to and during the periods of time that each nation or region has already identified as its RSV season. Once initiated, active surveillance systems should remain prioritized so that shifts in seasonality and/or meteorological influences on timing can be identified and changes in the timing of passive immunization adjusted accordingly. Implementing and/or continuing active surveillance carries the added benefit of the ability to estimate real world effectiveness of implementing any new or emerging RSV prevention strategy.

## Figures and Tables

**Table 1:** Respiratory syncytial virus (RSV) epidemiology in countries of tropical Asia.

Country	Reference	Study Years	Cohort Studied	Sample Size	RSV Positivity	Seasonality
Bangladesh	[16] ^a^	2015–2017	Malnourished children < 5 years ^g,j^	360	8.9%	All year round, peak December–February
Indonesia	[17] ^b^	2000–2002	<2 years ^g,j^	5187	20.1%	All year round, peak February–May
Indonesia	[18] ^b^	2000–2001	<2 years ^g,j^	2677	23%	All year round, peak February–May
Malaysia	[19] ^c^	Unknown	1 month–2 years ^g,j^	412	22.6%	All year round, peak September–December
Malaysia	[20] ^c^	2017–2018	>30 days old ^g,j^	600	20.4%	All year round
Malaysia	[21] ^c^	2009	1 month–5 years ^g,j^	165	50%	Throughout study period
Malaysia	[22] ^d^	1982–2008	<5 years ^g^	10,269	18.9%	Peak September–December
Nepal	[23] ^b^	2011–2014	6 months and older ^h,I,j^	1730	12.4%	Did not discuss
Nepal	[24]	2017–2018	‘children’	4252	19%	All year round, peaks July-August and January-February
Philippines	[25] ^d^	2008–2009	8 days–13 years ^g,j^	819	24.1%	September–December, peak October
Philippines	[26] ^e^	2008–2016	8 days to 5 years ^g,j^	5054	27%	Did not discuss
Philippines	[27] ^f^	2014–2016	<5 years ^h,I,j^	3851	12%	Did not discuss
Qatar	[28] ^d^	2012–2017	<15 years ^h,I,j^	33404	19.7%	All year round, peak November–December
Singapore	[29] ^d^	2011–2016	‘children’ ^g,I,j^	97840	23.8%	May to September
Sri Lanka	[30] ^c^	2015–2016	<5 years ^g,j^	818	27.6%	Not discussed
Sri Lanka	Divarante abstract	2016–2018	<5 years	502	32%	Not discussed
Sri Lanka	[31]	2013–2014	1 month–5 years	861	20.7%	Did not discuss
Thailand	[32] ^d^	2012–2018	5 years old and younger ^h,I,j^	8209	13.2%	July–November
Thailand	[33] ^b^	2010–2015	All individuals ^g,j^	972	18.7%	Did not discuss
Vietnam	[34] ^b^	2010	<16 years ^g,j^	1439	26%	April–December, July to October

^a^ Prospective case-controlled study; ^b^ Surveillance study; ^c^ Prospective cross sectional study; ^d^ Retrospective study; ^e^ Case series; ^f^ Prospective cohort study; ^g^ Hospitalized; ^h^ ambulatory; ^I^ upper respiratory symptoms; ^j^ lower respiratory symptoms.

**Table 2:** RSV epidemiology in countries of tropical Africa.

Country	Reference	Study Years	Age Group	Sample Size	RSV Positivity	Seasonality
Burkina Faso	[51] ^c^	2010–2011	<3 years ^g,h,I,j^	209	10.5%	Epidemics September–October
Cameroon	[52]	2018	<2 years	100	33%	Did not discuss
Cote d’Ivoire	[53] ^b^	2009–2010	<5 years ^h,I,j^	470	24%	Did not discuss
Ethiopia	[54]	2015–2016	Not noted	422	12.8%	Did not discuss
The Gambia	[55] ^b^	2015	2–23 months ^g,h,j^	519	47%	All year round
Ghana	[56]	2015–2016	<5 years	176	11.4%	Did not discuss
Ghana	[57]	2013–2014	<5 years	552	23%	June–December
Kenya	[58] ^b^	2009–2010	Households with infants ^h,i,j,k^	16,928	3.2%	January–May, peak March
Kenya	[59] ^b^	2002–2004	<5 years ^h,I,j^	2143	7.7%	Did not discuss
Kenya	[60] ^c^	2006–2018	<5 years ^g,h,I,j^	31,722	14%	Varies by county
Kenya	[61] ^c^	2015	<2 years ^g,j^	234	8.1%	Did not discuss
Madagascar	[62]	2011–2018	<5 years	1613	30%	Peak: March
Madagascar	[63] ^d^	2011–2017	<5 years ^g,h,I,j^	671	43.5%	Did not discuss
Madagascar	[64] ^b^	2010–2013	<5 years ^g,j^	876	37.7%	Circulates year round, timing of epidemics varies
Madagascar	[65] ^c^	2010–2011	2–59 months ^h,I,j^	295	11.8%	Seasonal variation, increased cases November–February
Madagascar	[66] ^b^	2008–2009	All ages ^h,I,j^	313	21.2%	February–May, peak March
Malawi	[67] ^b^	2011–2014	3 months–14 years ^g,h,I,j^	2363	11.9%	Peaks January–March
Mozambique	[68] ^c^	1998–2000	<1 yr ^h,j^, <5 years ^g,j^	5635 1307	10.6% 8.6%	January–April, peak March
Nigeria	[69]	2018	<5 years	106	33%	Did not discuss
Réunion Island	[70] ^d^	2011–2012	All ages ^h,I,j^	222	2.7%	Detected only during summer months

^a^ Prospective case-controlled study; ^b^ Surveillance study; ^c^ Prospective cross sectional study; ^d^ Retrospective study; ^e^ Case series; ^f^ Prospective cohort study; ^g^ Hospitalized; ^h^ ambulatory; ^I^ upper respiratory symptoms; ^j^ lower respiratory symptoms; ^k^ asymptomatic.

**Table 3:** RSV epidemiology in countries of tropical South America.

Country	Reference	Study Years	Age Group	Sample Size	RSV Positivity	Seasonality
Brazil	[82] ^c^	2012–2013	< 2 years ^g,j^	507	40.2%	Peaks June–July and February–March
Brazil	[89] ^c^	2009–2013	All ages ^h,I,j^	696	10.1%	Not discussed
Brazil	[90] ^c^	2009–2013	6–23 months ^h,I,j^	560	24.8%	RSV A: peak August–January; RSV B: peak March–June
Brazil	[91] ^b^	2005–2012	<5 years ^g,h^	12,501	11.6%	Did not discuss
Colombia	[92] ^c^	2005–2006	<1 yr ^g,j^	717	30%	Year round, peaks vary by geographic location
Colombia	[93] ^d^	2015–2016	<2 years ^g,j^	417	46.3%	Peaks April–August, November–January
Colombia	[94] ^c^	2009–2013	<3 years ^g,j^	13,488	33.8%	All year round, peak March–May
Ecuador	[95] ^b^	2009–2016	All ages ^g,h,I,j^	41,172	9.5%	Peak March
Ecuador	[96] ^c^	2008–2010	2–59 months ^g,j^	406	39.2%	Peak February–April
Peru	[86] ^f^	2009–2011	<3 years ^h,I,j^	892	23%	Did not discuss
Peru	[83] ^c^	2009–2010	‘children’ ^g,h,I,j^	717	15.3%	Did not discuss
Peru	[97] ^f^	2009–2011	<3 years ^h,I,j^	4475	3%	Did not discuss
Suriname	[81] ^b^	2016–2017	All ages ^g,h,I,j^	1096	19.4%	All year round

^a^ Prospective case-controlled study; ^b^ Surveillance study; ^c^ Prospective cross sectional study; ^d^ Retrospective study; ^e^ Case series; ^f^ Prospective cohort study; ^g^ Hospitalized; ^h^ ambulatory; ^I^ upper respiratory symptoms; ^j^ lower respiratory symptoms; ^k^ asymptomatic.
